# Rapid evolution of chemosensory receptor genes in a pair of sibling species of orchid bees (Apidae: Euglossini)

**DOI:** 10.1186/s12862-015-0451-9

**Published:** 2015-08-28

**Authors:** Philipp Brand, Santiago R. Ramírez, Florian Leese, J. Javier G. Quezada-Euan, Ralph Tollrian, Thomas Eltz

**Affiliations:** Department of Animal Ecology, Evolution and Biodiversity, Ruhr University Bochum, Universitätsstrasse 150, D-44801 Bochum, Germany; Department for Evolution and Ecology, Center for Population Biology, University of California Davis, One Shields Avenue, 95616 Davis, USA; Departamento de Apicultura, Universidad Autónoma de Yucatán, Mérida, Mexico; Present address: Faculty of Biology, Aquatic Ecosystems Research, University of Duisburg and Essen, Universitätsstrasse 5, D-45141 Essen, Germany

## Abstract

**Background:**

Insects rely more on chemical signals (semiochemicals) than on any other sensory modality to find, identify, and choose mates. In most insects, pheromone production is typically regulated through biosynthetic pathways, whereas pheromone sensory detection is controlled by the olfactory system. Orchid bees are exceptional in that their semiochemicals are not produced metabolically, but instead male bees collect odoriferous compounds (perfumes) from the environment and store them in specialized hind-leg pockets to subsequently expose during courtship display. Thus, the olfactory sensory system of orchid bees simultaneously controls male perfume traits (sender components) and female preferences (receiver components). This functional linkage increases the opportunities for parallel evolution of male traits and female preferences, particularly in response to genetic changes of chemosensory detection (*e.g.* Odorant Receptor genes). To identify whether shifts in pheromone composition among related lineages of orchid bees are associated with divergence in chemosensory genes of the olfactory periphery, we searched for patterns of divergent selection across the antennal transcriptomes of two recently diverged sibling species *Euglossa dilemma* and *E. viridissima*.

**Results:**

We identified 3185 orthologous genes including 94 chemosensory loci from five different gene families (Odorant Receptors, Ionotropic Receptors, Gustatory Receptors, Odorant Binding Proteins, and Chemosensory Proteins). Our results revealed that orthologs with signatures of divergent selection between *E. dilemma* and *E. viridissima* were significantly enriched for chemosensory genes. Notably, elevated signals of divergent selection were almost exclusively observed among chemosensory receptors (*i.e.* Odorant Receptors).

**Conclusions:**

Our results suggest that rapid changes in the chemosensory gene family occurred among closely related species of orchid bees. These findings are consistent with the hypothesis that strong divergent selection acting on chemosensory receptor genes plays an important role in the evolution and diversification of insect pheromone systems.

**Electronic supplementary material:**

The online version of this article (doi:10.1186/s12862-015-0451-9) contains supplementary material, which is available to authorized users.

## Background

Olfaction allows animals to perceive volatile chemicals from the environment and is therefore essential for the detection and discrimination of food resources, predators, and conspecifics in a diverse array of taxa [1, 2]. In insects, intraspecific olfactory communication is predominantly based on the recognition of endogenous pheromones that are used to trigger a plethora of behaviors, including social interaction, mate choice, and mate identification [3, 4]. Chemosensory genes expressed in the peripheral sensory neurons of the insect antennae enable the detection of pheromone compounds, and thus are crucially important for the detection of olfactory cues with diverse ecological functions [5, 6]. Closely related species of insects often exhibit pheromone signals with minute quantitative and qualitative differences [7]. Thus, highly specialized and sensitive signal recognition systems are necessary for discrimination of conspecific individuals and co-occurring (sympatric) species. However, despite the relative importance of pheromone detection in the evolution of insect communication and speciation, the genetic mechanisms underlying the differentiation of pheromone recognition systems remain poorly understood [7, 8].

Recent work on reproductively isolated sympatric races of the European corn borer, *Ostrinia nubilalis*, has shown that divergence in pheromone recognition might be best explained by nucleotide substitutions in pheromone receptor genes [9]. Although this study lacked a direct test on candidate genes, it suggests that molecular divergence of chemosensory genes could have promoted the early differentiation of pheromone sensory tuning in these two species of moth. This mechanism has been put forward to explain the rapid evolution of pheromone communication systems in other sympatric sibling species of Lepidoptera [10, 11]. In fact, a single receptor mutation was shown to drive the early divergence in pheromone detection in *Ostrinia furnacalis*, a close relative to *O. nubilalis* [12]. However, it remains unclear whether these findings are broadly applicable to other insect taxa that rely on pheromone reception for sexual communication, mainly because few studies have examined the evolution of peripheral olfactory systems in closely related species. Additionally, other molecular mechanisms could lead to changes in the odor perception ability of a species, including changes in expression rates of genes involved in olfaction [13–15] and the evolution of the gene repertoire through gene duplication and gene loss [16, 17]. However, the relative impact of these two mechanisms on pheromone recognition of closely related species is less well understood.

The process of differentiation of communication channels requires concomitant shifts in both signal emission and signal perception [18]. Thus, a change in the pheromone detection pathway is expected to take place along with a shift in pheromone composition. Pheromone signals are usually synthesized metabolically *de novo* from relatively simple precursor building blocks [19]. Therefore, a shift in the chemical communication system of an insect lineage requires the correlated modifications of two independent pathways: the biosynthesis of pheromone compounds and the olfactory detection of such pheromone compounds [3, 12, 20]. Here we introduce an insect communication system in which a single pathway (olfactory) is responsible for changes in both signal production and signal detection.

Orchid bees (Apidae; Euglossini) are some of the most important pollinators in the neotropical region, where they pollinate thousands of plant species from numerous angiosperm families [21]. Unlike other insects, male orchid bees utilize unmodified exogenous volatiles to communicate species affiliation [22–27] that are further hypothesized to address females in the context of mating (*e.g.* [26]). Male orchid bees collect chemical substances from various floral and non-floral sources to concoct a species-specific perfume blend [28–32]. The perfume is stored in specialized pouches in the bee’s hind-tibiae and is eventually released in a ritualized courtship display behavior at sites where females arrive for mating [30, 33–35]. Accordingly, it is expected that both male and female orchid bees use overlapping gene sets to detect perfume compounds (*e.g.* [27]). As a result, orchid bees rely on their olfactory sensory system to produce and detect species-specific chemical signals.

Although the precise physiological mechanisms of perfume discrimination remain unknown, previous studies indicate that the olfactory periphery (*i.e.* the antennal processes involved in translating chemical odor signals into neurophysiological responses) plays a critical role in compound discrimination. Preferences for conspecific perfume blends are accompanied by increased antennal responses in comparison to responses to perfumes of closely and distantly related species [25, 27]. Furthermore, single compounds that exclusively occur in the bouquet of a given species can elicit higher antennal responses of conspecifics compared to individuals of closely related species [27, 36]. This suggests the presence of chemosensory genes that are tuned towards key perfume compounds, similar to what has been described for lepidopteran pheromone receptors (*e.g.* [12]).

All insect pheromone receptor genes characterized to date, including the honeybee queen pheromone receptor, belong to the Odorant Receptor (OR) gene family, the largest of three chemosensory receptor multi-gene families involved in insect odor detection [6, 37–40]. Unlike ORs, only a subset of Ionotropic Receptors (IRs) and few members of the Gustatory Receptor (GR) gene family are associated with olfaction [41–43]. In addition to these three receptor gene families, Odorant Binding Proteins (OBPs) and Chemosensory Proteins (CSPs) play a crucial role in peripheral olfactory recognition [6, 44]. These two non-receptor multi-gene families encode soluble globular proteins that presumably help transport hydrophobic odorant molecules through the hydrophilic sensillum lymph [44]. While CSPs were shown to be involved in nest mate recognition in ants [45], OBPs are necessary for pheromone reception in different insect species [46–48]. The involvement of these five gene families in insect olfaction makes them potential targets of selection with cascading effects on intraspecific communication channels.

In this study we provide an analysis of the chemosensory gene families of two recently diverged (~0.15-0.11 mya) sibling species of orchid bees, namely *Euglossa dilemma* Bembé & Eltz and *E. viridissima* Friese from the Yúcatan Peninsula of Mexico [49]. The perfume profiles of these two morphologically [49] and ecologically [50] similar species differ mainly in the presence of a single compound (2-hydroxy-6-nona-1,3-dienyl-benzaldehyde, hereafter HNDB) in *E. dilemma* and its complete absence in *E. viridissima* perfumes [27, 49]. This compound of unknown origin is found in the perfume of only one other orchid bee species, namely *Euglossa mixta*, which is distantly related to *E. dilemma*, suggesting possible multiple independent origins of HNDB collection in orchid bees [32]. In fact, HNDB comprises on average more than 60 % of the *E. dilemma* perfume blend. Moreover, HNDB attracts volatile-seeking males of *E. dilemma* but not *E. viridissima* when presented as a single compound in chemical bioassays in the field [27]. Concordantly, antennae of both male and female *E. dilemma* are more sensitive to HNDB than those of *E. viridissima* suggesting that behavioral differences might be based on divergence in the antennal periphery ([27]; T. Eltz, unpublished). The marked recent divergence between these sibling species provides unique opportunities to study the evolutionary genetic mechanisms that shaped peripheral olfactory recognition systems. For this purpose, we first identified the repertoires of all five focal chemosensory gene families in *E. dilemma* and *E. viridissima*. Due to the large genome sizes (~4Gb, each; Ramírez et al. unpublished data) we employed an antennal transcriptome sequencing approach coupled with a highly conservative *de novo* meta-assembly strategy. We analyzed the orthologous chemosensory gene sets found in both species and screened for patterns of divergent evolution. Our findings demonstrate that, despite the overall low divergence between these sibling species, divergent evolution of key chemosensory genes is accelerated, possibly due to divergent selection on the OR gene family.

## Results

### Candidate gene detection

To identify chemosensory genes of the OR, IR, GR, OBP, and CSP gene families in *Euglossa dilemma* and *E. viridissima*, we reconstructed the antennal transcriptomes for each species using a conservative meta-assembly approach (see Methods). In order to validate assembly quality, we annotated the transcriptomes by BLAT comparisons to 10,602 honeybee reference protein sequences that are not members of the focal chemosensory gene families (non-chemosensory (NC) gene set; RefSeq database accessed 10/22/12). This resulted in 8710 unique annotations of which more than 90 % were detected independently in both species and >70 % of all shared annotations showed ≥95 % completeness and contiguity of open reading frames (ORFs) (see Additional file 1: Table S1). Overall, 3091 full-length ortholog NC genes passed further conservative filter criteria and were later used for divergence analyses.

Chemosensory gene discovery using iterative tBLASTn searches on the antennal transcriptomes revealed 117 *Euglossa* loci homologous to known members of the five targeted chemosensory gene families of bees and wasps (Additional file 1: Table S4) of which 95 (81 %) full-length orthologs were shared between *E. dilemma* and *E. viridissima* and thus were supported by independent discovery in the two sibling species (Table 1).

**Table 1:** Chemosensory genes detected in the antennal transcriptomes of *E. dilemma* and *E. viridissima*

	ORs	GRs	IRs	OBPs	CSPs	Total
*E. dilemma* ^a^	86 (5|3)	2 (1|1)	4 (0|0)	10 (0|0)	5 (0|0)	107 (6|4)
*E. viridissima* ^a^	85 (4|3)	4 (3|1)	5 (1|0)	11 (1|0)	6 (1|0)	111 (10|4)
Unique genes	90	5	5	11	6	117
Full-length orthologs^b^	75 (0.83)	1 (0.20)	4 (0.80)	10 (0.91)	5 (0.83)	95 (0.81)

^a^In brackets: unique genes found in only one of the two species | number of genes with missing N or C terminus

^b^Amount of full-length orthologs per gene family present in the antennal transcriptomes of both sibling species given in brackets

With 90 candidate genes, the largest number of loci we identified among chemosensory genes belonged to the OR gene family, corresponding to 77 % of all candidate chemosensory loci detected. Of all OR genes, 75 (83 %) were present in full length in the transcriptomes of both species. Additionally, of five candidate GRs and five candidate IRs, one and four orthologs were shared between species, respectively. The non-receptor chemosensory gene families were represented by 17 unique candidate genes, of which only two could not be detected in both species’ antennal transcriptomes. This resulted in 10 and five shared full-length ortholog OBPs and CSPs, respectively.

We note that it is unlikely that the detected candidate genes represent the complete repertoire of the *E. dilemma* and *E. viridissima* chemosensory gene families, because detection is not possible if expression levels of target genes are too low, or specific to unexamined sexes, life stages or tissues. For example, it has been established that there is a typical 1:1 relationship of ORs and the number of glomeruli in the antennal lobes across insect lineages [51, 52]. Thus, based on ~160 glomeruli in the antennal lobes of each analyzed *Euglossa* species (Ramírez and Eltz unpublished), we estimate that we detected ~50 % of the functional ORs. However, the detected genes likely cover a large fraction of chemosensory genes important in intraspecific olfactory communication, as these are typically among the highest expressed chemosensory genes in insects and thus very likely to be detected in antennal transcriptome analyses [11, 40, 53].

### Chemosensory gene family dynamics

**Fig. 1:**
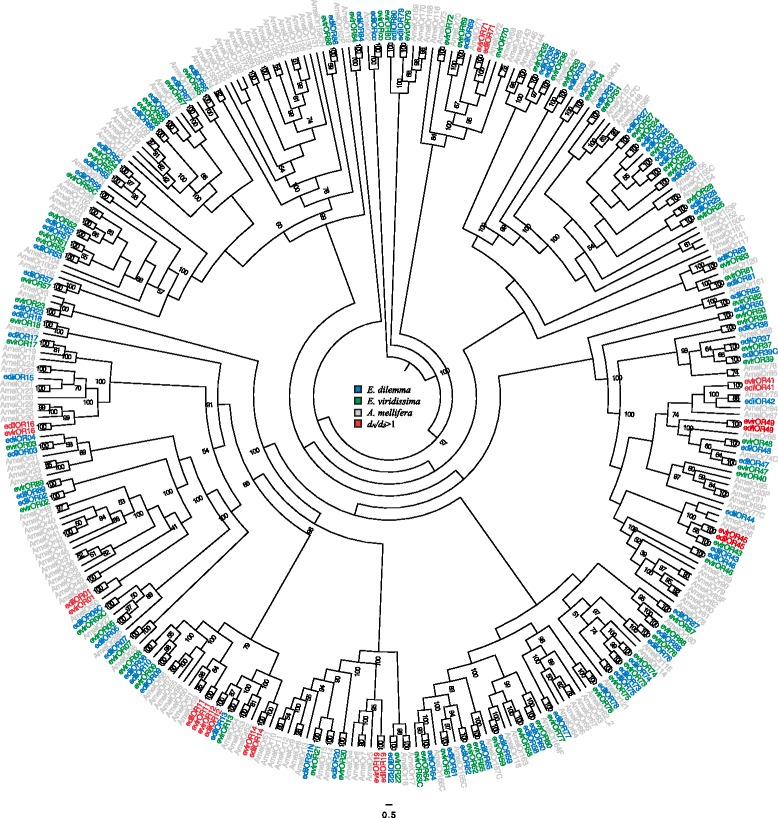
Phylogenetic relationships of the 86 candidate *E. dilemma* and 85 *E. viridissima* Odorant Receptors (ORs) to *Apis mellifera*. The maximum likelihood tree was rooted by the OR co-receptor orthologs of all three species. Bootstrap values for branches with ≥50 % support are indicated. ORs marked in red correspond to the 10 orthologs with *d*_*N*_ */d*_*S*_ > 1 (Table 2; see main text for details). Symbols after the OR descriptors: C: C-terminus is missing, N: N-terminus is missing, F: gene model was manually assembled, P: pseudogene (after [54])

Phylogenetic inferences of the candidate chemosensory genes validated the homology of all the loci detected in relation to their respective gene families. Each *Euglossa* locus clustered with known honeybee representatives of the assigned gene family (Fig. 1; Additional file 2: Figures S1-S4). In contrast to the OR gene family, we identified simple 1:1 orthologous relationships for all but two non-OR chemosensory genes to known chemosensory genes of the honeybee, the closest relative with a completely known chemosensory gene family set [54]. This included all five IRs, all of which were orthologous to genes of the olfactory ‘antennal IR’ subfamily ([42, 55]; Additional file 2: Figure S1). Of five GRs, four had simple orthologous relationships to known honeybee GRs including an ortholog of a candidate sugar receptor (EvirGR04). Interestingly, the GR without a simple ortholog formed the outgroup of a cluster of three honeybee GR pseudogenes (Additional file 2: Figure S2). Simple orthologs were also identified for all CSPs. All but one OBP (Additional file 2: Figure S3-S4) were also assigned to honeybee orthologs including one ortholog of OBP3 of the mason bee *Osmia cornuta* for which binding affinities to several odors have been established [56]. All the *Euglossa* OBPs we identified were found to be orthologous to OBPs of the classical subfamily and/or exhibited six conserved cysteines typical for that subfamily (Additional file 2: Figures S3 and S5). Interestingly, two candidate OBPs were orthologous to honeybee OBPs that are not expressed in antennal tissue of either sex or caste, and were also absent in antennal transcriptomes of *O. cornuta* [56, 57].

The relationships between the ORs of *Euglossa* and the honeybee were less unambiguous in relation to the dynamics of the other chemosensory families. Only 28 of the 90 detected *Euglossa* ORs (31 %) showed simple 1:1 orthology to known honeybee ORs (22 of these with ≥95 % bootstrap support, Fig. 1), indicating increased gene family divergence. Although the *Euglossa* homolog of the highly conserved OR co-receptor [58, 59] could be identified, none of the *Euglossa* ORs were orthologous to any of the three honeybee ORs that have been functionally characterized to date [38, 60]. Thus, potential ligands of *Euglossa* ORs remain unidentified. The lack of orthology of several *Euglossa*-specific ORs to honeybee ORs, indicated 10 *Euglossa*-specific OR duplications signified by clusters of one honeybee OR as outgroup to two different *Euglossa* ORs (Fig. 1; *e.g.* AmelOR114 to evir/edilOR73-74). We furthermore found two subfamily expansions of three or more *Euglossa* ORs (Fig. 1; *e.g.* evir/edilOR08-10). On the other hand, several honeybee ORs had no direct orthologs that could be detected in *Euglossa*. This is not surprising given the likely incompleteness of OR sets in the analyzed species of *Euglossa* (see above).

### Patterns of nucleotide polymorphism and diversifying selection

We mapped antennal reads from a pool of 40 haploid males for each species against the ORFs of all the detected full-length chemosensory and NC genes. We excluded ORFs that lacked a mean per-base coverage ≥10-fold in both orthologs, but retained 3091 NC genes as well as 74 ORs, four IRs, one GR, 10 OBPs and five CSPs (Table 2). Overall, 42 chemosensory loci and 387 NC loci were variable (polymorphic) between *E. dilemma* and *E. viridissima*, corresponding to 45 % and 13 % of the reconstructed candidate genes, respectively. With 36 loci, the majority (86 %) of variable chemosensory genes belonged to the OR gene family, while for each remaining chemosensory gene family only one or two loci showed polymorphisms between the two species (Table 2). This is not surprising given the number of reconstructed loci in each gene family. In total, we identified 1207 variable sites, of which 218 were found in chemosensory genes and 989 in NC genes, with 101 (46 %) and 376 (38 %) sites, respectively, representing fixed differences between *E. dilemma* and *E. viridissima*. Altogether, 24 chemosensory loci (21 ORs, two IRs and one OBP) and 157 NC loci contained sites fixed for different nucleotides in the two species (Table 2). Overall, the ratio of fixed to polymorphic sites was higher in the chemosensory gene family sets (0.86 and 0.61 for chemosensory and NC genes, respectively; Fisher’s exact test; *p* = 0.02637). Furthermore, the chemosensory gene families showed a significantly elevated ratio of non-synonymous to synonymous fixed sites in comparison to the NC gene set (Table 2; Fisher’s exact test; *p* < 2.2e-16).

**Table 2:** Nucleotide polymorphisms and patterns of selection between orthologous genes

	ORs	GRs	IRs	OBPs	CSPs	Total	NC
Unique genes^a^	74	1	4	10	5	94	3091
Variable genes^b^	36 (0.49)	1 (1.0)	2 (0.5)	2 (0.2)	1 (0.2)	42 (0.45)	387 (0.13)
Total variable sites	194	3	13	10	1	218	989
Total polymorphic^c^							
synonymous	55	0	2	3	1	61	479
non-synonymous	50	3	2	4	0	56	134
Total fixed^c^							
synonymous	23	0	1	1	0	25	277
non-synonymous	66	0	8	2	0	76	99
Genes fixed^d^	21	0	2	1	0	24	157
Mean d_N_^e^	0.0034857	0	0.0026	0.006000	0	0.0035167	0.0005127
Mean d_S_^e^	0.0037524	0	0.00105	0.011600	0	0.0038542	0.0042777
Mean d_N_/d_S_^e^	0.9289340	-	2.4761905	0.5172414	-	0.9124324	0.1198630
d_N_/d_S_ > 1^e,f^	10 (1)	-	2 (0)	0 (0)	-	12 (1)	23 (0)
S ratio [%]^g^	13.51	0	50	0	0	12.77	0.74

^a^Homologous genes identified independently in the antennal transcriptomes of *E. dilemma* and *E. viridissima* with ≥10-fold mean per-base coverage

^b^Genes with fixed differences between the two species. In brackets: relative amount of all unique genes

^c^Total polymorphic/ fixed sites of all variable sites

^d^Genes with fixed differences between the two species

^e^ *d*_*N*_ */d*_*S*_ calculations are based on genes containing fixed differences

^f^Number of pairwise *d*_*N*_ */d*_*S*_ estimates significantly higher than 1 in brackets

^g^Ratio of orthologous genes with fixed differences among all orthologous genes detected per gene set

**Fig. 2:**
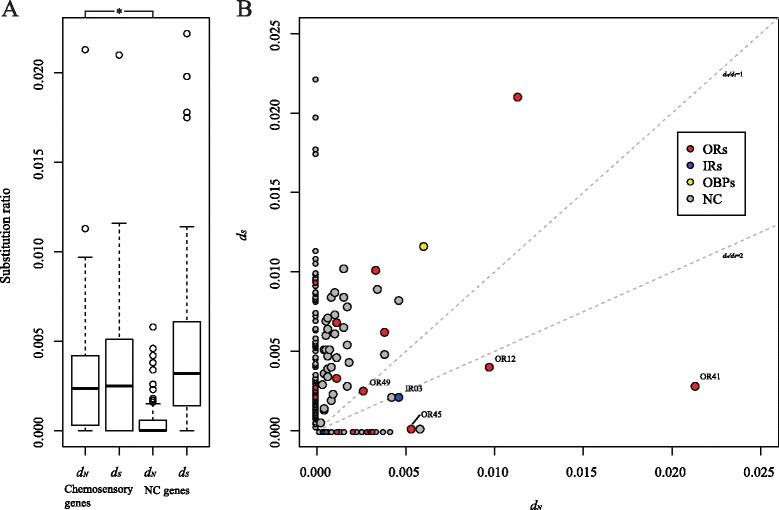
Analysis of divergent selection between *E. dilemma* and *E. viridissima*. **a** Boxplot comparing *d*_*N*_ and *d*_*S*_ values obtained for chemosensory and non-chemosensory (NC) genes (*d*_*N*_ and/or *d*_*S*_ ≠ 0). *d*_*N*_ was significantly higher for chemosensory than for NC loci while *d*_*S*_ had similar values for both sets resulting in elevated mean *d*_*N*_ */d*_*S*_ for the chemosensory loci (see text for statistics). *: *p* < 0.001. **b** *d*_*N*_ */d*_*S*_ plot for 3185 genes reconstructed from the antennal transcriptome analysis. Those genes exhibiting *d*_*N*_ */d*_*S*_ >1 have higher non-synonymous to synonymous substitution rates, in agreement with the hypothesis of divergent selection (lower right); those genes with *d*_*N*_ */d*_*S*_ <1 exhibit lower non-synonymous to synonymous substitution rates, being consistent with the hypothesis of purifying selection (upper left). Genes with zero *d*_*N*_ and *d*_*S*_ are not shown and genes with either *d*_*N*_ or *d*_*S*_ = 0 are indicated by small points. The set of genes with *d*_*N*_ */d*_*S*_ >1 was enriched for chemosensory receptor genes. ORs: Odorant receptors, IRs: Ionotropic receptors, OBPs: Odorant-binding proteins

Patterns of nucleotide polymorphisms enriched for fixed non-synonymous in comparison to synonymous substitutions as observed for the combined chemosensory gene set are expected in the presence of diversifying selection. To test for diversifying selection on the 181 candidate genes with fixed interspecific differences, we used the non-synonymous and synonymous substitution rates between *E. dilemma* and *E. viridissima* to calculate pairwise replacement to silent substitution rate ratios (*d*_*N*_*/d*_*S*_). Therefore, we only took fixed sites into consideration because polymorphic sites are known to inflate *d*_*N*_*/d*_*S*_ estimates, especially between species with comparatively low divergence times (see Methods; [61]). Consistent with the observed patterns of nucleotide polymorphisms, *d*_*N*_ was significantly higher for chemosensory loci than for NC loci (Mann–Whitney U = 2933; *p*-value = 8.462e-7) while *d*_*S*_ showed similar values for both sets of genes (Mann–Whitney U = 1624; *p*-value = 0.2768). This resulted in an elevated mean *d*_*N*_*/d*_*S*_ for the chemosensory loci (0.91 vs. 0.12 for the NC gene set; Fig. 2a; Table 2) indicating relaxed purifying and/or increased diversifying selection on chemosensory genes. Although mean *d*_*N*_*/d*_*S*_ was smaller than 1 for both gene sets, we detected 12 chemosensory receptors (10 ORs and 2 IRs; Table 3) and 23 NC genes with *d*_*N*_*/d*_*S*_ > 1 (Fig. 2b; Table 2; Additional file 1: Table S2), indicating that positive selection may have driven divergence in these candidate genes. The set of genes corresponds to 12.8 % of the chemosensory loci identified and 0.7 % of the NC gene set. This observation suggests that divergent selective pressures are increased in genes of the olfactory periphery, in particular in chemosensory receptors (Fisher’s exact test; *p* = 3.843e-10). Accordingly, the set of genes with *d*_*N*_*/d*_*S*_ > 1 was significantly enriched for chemosensory receptors compared to those with *d*_*N*_*/d*_*S*_ < 1 (Fisher’s exact test; *p* = 3.92e-10).

**Table 3:** Fixed and polymorphic non-synonymous and synonymous substiutions of orthologous chemosensory genes with *d*_*N*_ */d*_*S*_ > 1

	Non-synonymous^a^		Synonymous^a^					
Gene	Fixed	Polymorphic	Fixed	Polymorphic	*d*_*N*_	*d*_*S*_	*d*_*N*_ */d*_*S*_^b^	In LBD^c^
OR41	18	1	1	5	0.0213	0.0028	7.7278*	9
OR12	10	6	1	2	0.0097	0.0040	2.4235	3
OR45	5	1	0	0	0.0053	0.0000	∞	5
OR14	3	2	0	4	0.0021	0.0000	∞	1
OR01	3	1	0	3	0.0031	0.0000	∞	2
OR16	2	5	0	1	0.0032	0.0000	∞	1
OR71	2	5	0	0	0.0029	0	∞	1
OR49	2	2	1	1	0.0026	0.0025	1.0536	2
OR11	1	0	0	1	0.0012	0	∞	0
OR19	1	0	0	0	0.0012	0	∞	0
IR03	7	2	1	1	0.0046	0.0021	2.2076	3
IR11	1	0	0	1	0.0006	0	∞	0

Genes with less than 3 fixed substitutions between the two species are highlighted in grey

^a^Fixed and polymorphic non-synonymous and synonymous substitutions between orthologs of given genes of *E. dilemma* and *E. viridissima*

^b^Receptors with *d*_*N*_ */d*_*S*_ significantly higher than 1 are indicated by *

^c^Fixed substitutions in ligand binding domains (LBD) of ORs (transmembrane regions) and IRs (S1 and S2 LBD)

We estimated a mean of 2.9 fixed substitutions per gene among the 35 genes with *d*_*N*_*/d*_*S*_ > 1 (101 fixed substitutions in 35 genes; median: 1). In fact, only 12 of these genes contained at least three fixed substitutions (seven out of 12 chemosensory genes (58 %), five out of 23 NC genes (22 %)). In addition, 20 of the 23 remaining genes had only 1 fixed substitution, of which the majority (85 %) belonged to the NC gene set (three chemosensory genes, 17 NC genes; Table 3; Additional file 1: Table S2). Thus, most of the genes with *d*_*N*_*/d*_*S*_ > 1 were fixed for just one non-synonymous substitution between *E. dilemma* and *E. viridissima.*

To test whether the *d*_*N*_*/d*_*S*_ values differ from a null model of neutral evolution, we applied likelihood ratio tests to all orthologous pairs of chemosensory and NC genes. Interestingly, of the 181 gene pairs with fixed interspecific differences, only two *d*_*N*_*/d*_*S*_ estimates were significantly different from the null model. Of these OR41 showed a *d*_*N*_*/d*_*S*_ significantly higher than 1 (*d*_*N*_*/d*_*S*_ = 7.73; Likelihood-ratio test ∆ = 7.09; *p* < 0.01; Table 2 & 3) and OR06 showed a *d*_*N*_*/d*_*S*_ significantly lower than 1 (*d*_*N*_*/d*_*S*_ = 0.001; Likelihood-ratio test ∆ = 5.62; *p* < 0.05; Additional file 1: Table S5). This low number of genes diverging from the neutral null model is likely to reflect the generally low power of pairwise *d*_*N*_*/d*_*S*_ estimates [62] as well as the low number of fixed differences between the two species.

### Spatial distribution of non-synonymous substitutions

**Fig. 3:**
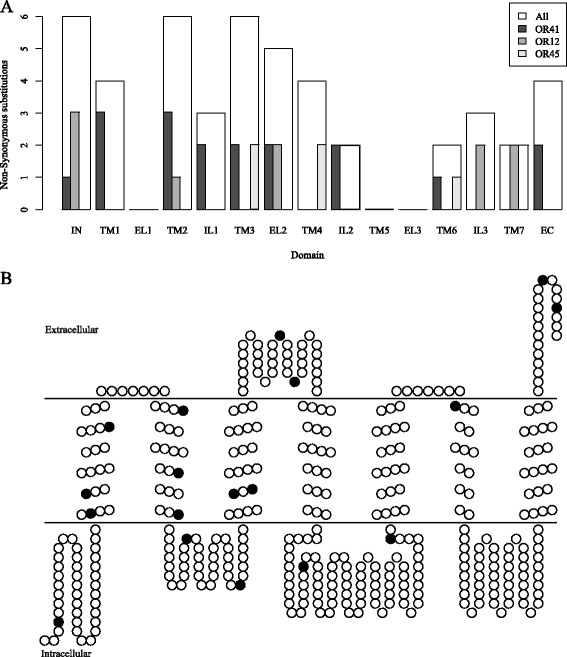
Distribution of non-synonymous amino acid substitutions across Odorant Receptor (OR) domains. **a** The white bars represent the sum of all non-synonymous substitutions detected in the respective domain over all ORs. OR12, OR41 and OR45 are highlighted because they showed the most non-synonymous substitutions between *E. dilemma* and *E. viridissima*. IN: Intracellular N-terminus, TM: Transmembrane domain, EL: External loop, IL: Internal loop, EC: Extracellular C-terminus. **b** Predicted membrane topology for OR41. Fixed non-synonymous substitutions between *E. dilemma* and *E. viridissima* are highlighted in black

Non-synonymous changes in ligand binding domains of receptor proteins can alter affinities towards ligands, modifying ligand interaction patterns [12, 63–67]. We determined the spatial distribution of non-synonymous substitutions along OR and IR protein sequences with *d*_*N*_*/d*_*S*_ > 1 to examine potential effects on ligand binding domains. Therefore, we predicted transmembrane domains (Additional file 1: Table S6; Additional file 2: Figure S6), the regions of OR proteins most sensitive to non-synonymous substitutions with regard to ligand binding [12, 64, 66]. Moreover, we used homology to known *Drosophila* IRs and the closely related ionotropic glutamate receptors (iGluRs) to infer ligand-binding domains (see Methods). In total, 24 (51 %) of the 47 non-synonymous substitutions fixed in the 10 ORs having *d*_*N*_*/d*_*S*_ > 1 between *E. dilemma* and *E. viridissima* were located in one of the seven transmembrane domains (Table 3; Fig. 3) which covered between 19.3 % and 35.8 % of the OR amino acid sequence (Mean: 30.5 %; Additional file 1: Table S6). Additionally, 3 (38 %) of 8 replacement substitutions were located in the IR ligand binding domains that covered 16.5 % and 14.7 % of the IR03 and IR11 amino acid sequence, respectively (Mean: 15.6). Interestingly, only three of the 12 chemosensory receptors (two ORs and one IR) did not reveal any change in the amino acid sequence of respective ligand binding domains. In order to test whether fixed non-synonymous substitutions are randomly distributed among ORs and IRs, we applied a goodness-of-fit test on the observed number of substitutions by estimating the mean proportion of receptor proteins that span ligand-binding domains. These tests revealed that the observed number of fixed non-synonymous substitutions were non-randomly distributed among ORs and were significantly enriched for transmembrane domains (Goodness-of-fit *χ*^2^ = 9.38; *p* < 0.01; IRs: *χ*^2^ = 2.91; *p* < 0.1). Furthermore, non-synonymous substitutions in ligand-binding domains were positively correlated with the number of non-synonymous substitutions (Pearson’s correlation coefficient; r = 0.91; *p* < 0.001). Concomitantly, the four chemosensory receptors that exhibited at least five fixed substitutions had the most replacement substitutions in ligand binding domains (up to 9 in OR41; Table 3), thus increasing the likelihood that such non-synonymous substitutions lead to changes in ligand-binding affinities.

Graphical analysis of the spatial distribution along the ORs revealed that the majority of non-synonymous substitutions are located towards the N-terminus rather than towards the C-terminus of the receptor (Fig. 3), matching known patterns of higher OR sequence conservation towards the C-terminus [68–70]. This pattern is also retained for a subset of the three ORs with at least five fixed substitutions between *E. dilemma* and *E. viridissima*.

## Discussion

The differentiation of intraspecific chemical communication systems depends on correlated shifts of signal production and signal detection [7]. In the case of chemical signaling of insect species, the emitter and receiver components are typically controlled by independent genetic pathways [18]. In the present study we focused on an exceptional communication system, where a single pathway regulates both signal production and signal detection. Orchid bees rely on their peripheral olfactory system to detect compounds for the composition of their so-called perfumes. These perfume phenotypes are thought to function as intraspecific recognition signals and have been shown to be highly differentiated even among closely related species [25–27, 32]. As a result, chemosensory genes are likely the targets of selection during the evolutionary process of signal differentiation. We tested this hypothesis in a pair of recently diverged sibling species of orchid bees, *Euglossa dilemma* and *E. viridissima* [49] whose perfumes differ mainly in one key chemical component. We compared the evolutionary patterns of differentiation of 94 orthologous chemosensory genes and 3091 genes that are not involved in chemoreception (NC genes) derived from a *de novo* antennal transcriptome analysis.

### Accelerated evolution of the olfactory periphery

Comparisons of the estimated *d*_*N*_ and *d*_*S*_ patterns based on orthologous chemosensory and NC genes indicate faster evolution of the chemosensory gene families driven in part by strong divergent selective pressures. Our analysis identified fixed nucleotide differences between the two sister species in 13 % of all analyzed genes. This low percentage probably reflects the short time span since the divergence of *E. dilemma* and *E. viridissima* [49]. However, chemosensory genes exhibited an elevated mean non-synonymous substitution rate (*d*_*N*_) that produced a mean *d*_*N*_*/d*_*S*_ ratio ~7.5 times higher than those estimated for NC genes. While this pattern is consistent with accelerated diversification rates in the chemosensory gene families, several underlying mechanisms could be at play. First, this pattern could be explained by relaxed purifying selection on chemosensory genes. Relaxation of purifying selection in chemosensory genes is usually expected following gene duplication events [71, 72]. However, analyses of the OR gene families from a number of *Drosophila* species have indicated that lineage-specific duplications among closely related sibling species (<10 mya divergence time) are rare [73, 74]. Although the OR gene family is the most dynamic among all chemosensory gene families, previous studies have identified very few lineage-specific duplication events, even among species evolving under highly divergent ecological conditions. For instance a maximum of four duplication events was estimated between the host specialist *D. sechellia* and the host generalist *D. simulans*, which have a divergence time of ~0.5 mya [73, 74]. In contrast, the elevated *d*_*N*_*/d*_*S*_ ratio that we observed between *E. dilemma* and *E. viridissima* is based on 42 variable chemosensory genes including 36 variable ORs. Hence, it is unlikely that the observed patterns are greatly influenced by lineage specific duplication events. Consistently, we could not find any evidence for lineage specific duplications in either species. However, we note that recent duplication events may have gone undetected in our dataset for several reasons (*e.g.* low expression levels) and that relaxation of purifying selective pressures might occur even in the absence of duplication events. We therefore cannot exclude relaxed purifying selection as a mechanism that shaped the evolution of chemosensory genes in orchid bees.

Alternatively, the observed patterns of *d*_*N*_*/d*_*S*_ ratios could reflect signatures of positive selection. We identified 12 chemosensory genes with *d*_*N*_*/d*_*S*_ > 1, which is consistent with the hypothesis of positive selection in one or both *Euglossa* lineages. Nevertheless, due to the overall low variability between species, the calculated *d*_*N*_*/d*_*S*_ ratios for several loci were based on few fixed differences alone. While previous studies have reported relatively low variability in chemosensory genes under divergent selective pressures in closely related insect species [75], we detected genes with both *d*_*N*_*/d*_*S*_ > 1 and comparably high interspecific diversity of which one (OR41) was significantly different from a neutral null model of sequence divergence (Table 2). The fact that only a single gene exhibited a significantly elevated *d*_*N*_*/d*_*S*_ ratio is not surprising given the low amount of fixed interspecific differences between *E. dilemma* and *E. viridissima* and the low power of pairwise *d*_*N*_*/d*_*S*_ tests in detecting genes under divergent selection [62, 76]. Accordingly, we expect that the test produced several false negatives that may be revealed by more sensitive phylogeny-based *d*_*N*_*/d*_*S*_ tests, as well as more comprehensive lineage sampling [62]. In addition, the comparatively short divergence time between *E. dilemma* and *E. viridissima* bears the potential of biasing *d*_*N*_*/d*_*S*_ estimates, that could lead to false positives [61, 77]. To account for this, we chose an approach that enabled the detection and exclusion of the main source of *d*_*N*_*/d*_*S*_ inflation, namely segregating polymorphisms [61, 77]. The resulting patterns of *d*_*N*_ and *d*_*S*_ that we observed between chemosensory genes and the NC gene set (see above) using fixed differences only are similar to that observed in other insects that exhibit much greater divergence times [16], thus indicating that our approach is suited for the detection of selective pressures on the chemosensory gene sets of *E. dilemma* and *E. viridissima*.

Our results are consistent with the hypothesis that genes of the olfactory peripheral system of *E. dilemma* and *E. viridissima* have evolved under strong divergent selective pressures. Together, these observations support a significant trend of increased divergent selective pressures that may have shaped the recent evolution of chemosensory genes in *E. dilemma* and *E. viridissima*.

### Are the observed patterns of diversifying selection related to divergence in chemical signaling?

The observed sequence divergence in orthologous olfactory receptor genes of *E. dilemma* and *E. viridissima* is likely linked to differences in the sensory tuning of each species, and possibly represents a response to divergent selection on chemosensory traits [7]. Various ecological factors may have promoted such differentiation, including host shifts [78, 79] and changes in mating ecology [12]. In solitary bees, several ecological factors may impose selective pressures on sensory detection, including foraging on different food resources (generalist vs. specialist), the use of different nesting materials, and the detection of suitable mating places and partners. However, both *E. dilemma* and *E. viridissima* are pollen generalists that are known to use very similar food resources [50], and the two species lack any noticeable differences in nesting biology (T. Eltz and S. Ramírez, pers. obs.). Thus, it is unlikely that the divergence we observed in olfactory receptor genes was due to selective pressures acting on foraging specialization. The most pronounced difference that has been documented between the two sympatric sibling species is on the chemical composition of male perfumes [27, 49].

The males of all species of orchid bees collect and accumulate species-specific perfumes [32] from a variety of sources. Likewise, *E. dilemma* and *E. viridissima* show distinct species-specific perfume phenotypes that are qualitatively and quantitatively differentiated. In particular, these two species differ in the presence of HNDB in *E. dilemma* perfumes and the complete absence in *E. viridissima* [27, 49]. This difference corresponds to pronounced behavioral and physiological responses. Males of *E. dilemma* are strongly attracted to HNDB, whereas males of *E. viridissima* are never attracted to this compound [27]. Correspondingly, the antennae of *E. dilemma* exhibit a significantly stronger neurophysiological response and sensitivity to HNDB, compared to a weaker response in *E. viridissima* [27]. Consequently, the observed divergence of the chemosensory gene families on the molecular level suggest that the chemical, physiological, and behavioral differences between this pair of species might be mediated—at least partially—by some of the genetic differences we identified in the chemosensory gene families.

Previous studies have hypothesized that perfume bouquets in orchid bees function as species-specific signals that are addressed to conspecific females in the context of mating (see Discussion in [26]). Thus, because olfaction determines both signal production and signal detection, evolutionary shifts in the olfactory pathway may lead to concomitant divergence in sexual communication in orchid bees. Genomic studies on closely related species of drosophilid flies have revealed that the primary targets of diversifying selection are, in fact, sex-related genes [80]. Moreover, previous studies on Lepidoptera and drosophilid flies have determined that those OR genes with elevated *d*_*N*_*/d*_*S*_ ratios tend to be involved in the perception of sex pheromones [12, 13]. Our results lend support to the hypothesis that candidate chemosensory receptors evolved under strong divergent selection in the *E. dilemma* and *E. viridissima* lineage, and thus selective forces may have contributed to shifts in the detection of odors that mediate sexual communication in one or both species.

### Chemosensory receptor driven evolution of the peripheral olfactory pathway

The observed pattern of divergent selection in chemosensory receptors suggests that the olfactory peripheral system plays a major role in the evolution of olfactory specialization in *E. dilemma* and *E. viridissima*. Among all the five families of chemosensory genes we studied, signatures of divergent selection were only present in OR genes and antennal IR genes. This result is partially consistent with an earlier hypothesis that a shift in peripheral olfaction between *E. dilemma* and *E. viridissima* might be driven by molecular divergence in OR genes [27], a common mechanism of olfactory diversification in insects [12, 64, 65].

Currently we lack information on the functional properties of the detected divergent chemosensory receptors. To gain some insight into potential impact of the observed substitutions, we inferred the transmembrane (TM) and ligand-biding domains in both ORs and IRs. Based on these topological predictions, the majority of the molecular differences between the sister species could affect the biochemical features of the receptors. For most of the ORs that exhibited signatures of diversifying selection, amino acid substitutions were detected in TM regions, where a single replacement can be sufficient to elicit shifts in ligand binding properties, as previously demonstrated in several insect taxa [12, 64–67]. Predicted amino acid substitutions in the S1 and S2 binding domains of a candidate IR might have similar consequences [42, 81, 82]. Accordingly, diversifying selection acting on *Euglossa* ORs and IRs might have led to divergent ligand binding properties of chemosensory receptor orthologs of the two species and in turn could account for the observed differences in antennal responses.

The neurophysiological difference in the response of *E. dilemma* and *E. viridissima* to volatile compounds could also be determined by higher-level integration of the central nervous system, *e.g.* neuronal networks in the antennal lobe or mushroom body. Nevertheless, the strong differentiation observed in antennal responses supports a scenario where the olfactory peripheral system profoundly affects the functioning of this chemical sexual communication system [27]. In orchid bees, differentiation of olfactory tuning through divergence of chemosensory receptors could have cascading effects on the species-specific perfumes that male bees acquire. Similarly, studies on moths have identified single non-synonymous substitutions in OR genes that lead to strong differentiation in pheromone detection abilities [9, 12, 14]. Comparative analyses have shown that the perfume phenotypes of orchid bees evolve exceptionally fast, even among closely related species [32]. Because non-synonymous substitutions in the olfactory periphery may simultaneously affect both male traits and female preference, diversifying selection acting on chemosensory receptors could serve as a mechanism to account for the fast evolutionary rates observed in perfume phenotypes among orchid bees. In fact, physical genetic linkage between sender and receiver genes could potentially accelerate the evolution of assortative mating and rapid modification of sexual communication channels (*e.g.* [83]). This, in turn, could help explain the high species richness of orchid bees throughout the neotropical region [84], and could lead to an understanding of the speciation mechanisms in orchid bees.

## Conclusions

While the vast majority of insect species relies on chemical communication to find mates in order to successfully reproduce, the genetic mechanisms underlying the evolution of pheromone recognition systems remain poorly understood, especially for non-model organisms. In this study we show that gene families in the olfactory periphery of *Euglossa dilemma* and *E. viridissima*, two recently diverged orchid bee species, likely evolved under divergent selection. Because signal production and signal detection are genetically linked in orchid bees, our findings support the hypothesis that divergent evolution of OR genes likely played a role in shaping both olfactory perception and divergence of chemical mating signals. Our results are consistent with previous studies on lepidopterans and indicate the general significance of selection acting on chemosensory receptors as a driver mechanism of diversification in insect pheromones.

## Methods

### Sampling and sequencing

Males of the two orchid bee species *Euglossa dilemma* and *E. viridissima* were sampled in the Yucatán Peninsula, Mexico in October 2011 near the city of Xmatkuil and between Muna and Uxmal (distance to Xmatkuil: ~50 km) using different chemical baits [85]. Sampling was performed with the necessary permits issued by the Secretaría de Medio Ambiente y Recursos Naturales to J.J.G. Quezada-Euan. Bees were kept in small cages in a greenhouse (temperature 20–24 °C). During the two to eight days in captivity a 1:3 mixture of honey and tap water was provided as food source. To produce antennal transcriptomes representing Yucatán populations, the antennae of 40 male specimens of each species were pooled for RNA-extraction. Bees were chilled on ice and the antennae of each torpid male were dissected by sterile forceps and immediately shock-frozen on liquid nitrogen. Antennae were kept on liquid nitrogen/dry ice until RNA-extraction.

Total-RNA was extracted using the TRIzol extraction method (Invitrogen) following the manufacturers tissue preparation protocol, except for an extended incubation time of 15 min in the phase separation step to maximize RNA yield. Extracted RNA was resuspended in 30 μl of RNase free water. All optional steps were skipped. RNA pools were treated with DNaseI to purge potential DNA contamination and subsequently quantified on the Experion Automated Electrophoresis System (Bio-Rad) with the Experion StdSens Analysis Kit (Bio-Rad) according to the standard protocol. Afterwards, 4 μg and 2 μg of total-RNA of the *E. dilemma* and *E. viridissima* pool, respectively were sent to GATC-Biotech (Constance, Germany) for barcoded cDNA library preparation using the TruSeq mRNA kit (Illumina) and subsequent 100-bp single-end sequencing on an Illumina HiSeq 2000 lane (Raw sequence reads are available at the NCBI Sequence Read Archive [SRA: SRX765918, SRA: SRX765888]).

### Pre-processing

Identical raw reads were merged using Fulcrum 0.4.2 [86] to improve assembly quality and computing efficiency [87]. The merged read sets were quality checked and reads were trimmed on both sides if sequencing primers or low-quality bases (Phred-score ≤ 20) were detected, applying a sliding window approach in SeqtrimNext [88] with a window size of 3. Furthermore, homopolymeric reads and reads < 21 bp were discarded using an inhouse Perl-script.

### Assembly and transcript recovery

Due to a lack of comprehensive sequence data for orchid bees, the antennal transcriptomes had to be assembled *de novo*. As the scope of this study required accurate reconstruction of candidate olfaction related ORFs, a thorough validation process was utilized. To minimize the probability of annotating misassembled transcripts, a meta-assembly-like approach was chosen (*cf.* [89, 90]).

### *De novo* transcriptome assembly

The pre-processed reads of each species were assembled using the two *de novo* transcriptome assemblers Trinity release 2012-03-17 [91, 92] and Velvet v1.2.04/Oases v1.2.03 [93, 94]. Since different assembler settings show different performances in transcript reconstruction (Additional file 1: Table S1; P. Brand, pers. obs.), the assemblers were run with nine different combinations of two different parameters controlling the threshold for contig elongation in respect to overall contig-coverage. The chosen combinations represent a range from relaxed to conservative settings for both assemblers. In these combinations Parameter 1 was set to 3, 5 or 7 and Parameter 2 to 0.05, 0.10 or 0.33, where Parameter 1 refers to the -cov_cutoff and --min_glue parameters, and Parameter 2 to the -edgeFractionCutoff and --min_iso_ratio parameters of Oases and Trinity, respectively. Applying all possible combinations of the two parameters for each assembler on both species’ read sets resulted in 18 assemblies per species. Trinity was always run with the default k-value of 25 while Oases was used in multiple-k-mer mode [95] with k-values of 21, 23, 25, 31, 37, 43, 49, 59, 69, 79 and 89 that were merged with a k-value of 27 using the merge mode of Oases. Minimum contig length was set to 100 bp for both assemblers.

### Detection of candidate transcripts

We applied standalone BLAT (BLAT-score threshold: 100, minimum sequence identity: 75 %; [96] to annotate all 36 transcriptome assemblies independently using 10,602 unique *Apis mellifera* Refseq proteins not involved in olfaction as reference (accessed 10/22/12; [97]). For each species, all annotations with a completeness and contiguity ≥ 95 % in at least one assembly of each assembler (see Additional file 1: Table S1 for differences in assemblies) were extracted, translated to the corresponding amino acid sequences and validated via BLASTp homology searches against the Refseq proteins. To find orthologous sequences present in both species sets, we used a reciprocal BLAST approach [98]. Two annotated open reading frames (ORFs) were considered orthologous when showing identical length and at least 95 % sequence identity. In fact, the number of orthologs did not increase when decreasing the minimum required sequence identity levels to as low as 50 % in any gene family (data not shown). Candidate orthologs were discarded when frameshifts or preliminary stop codons were present. All orthologs passing our filter settings were combined in the non-chemosensory gene set (NC genes).

To detect the maximum number of chemosensory genes we applied an iterative tBLASTn approach (*cf.* [53]). Homology searches were based on gene family specific query libraries comprised of published hymenopteran OBP, CSP, GR, IR and OR protein sequences [54–57, 99–101] (Additional file 1: Table S3). Transcripts with BLAST hits ≤ 1e^−06^ were searched for all possible ORFs ≥ 300 bp for OBPs and CSPs and ≥ 900 bp for the chemosensory receptors. Detected ORFs were validated via BLASTp homology searches against the respective query library and subsequently reused as queries to search for potentially undetected ORFs. This loop was repeated until no further ORFs of sufficient length and/or e-value were detected. The pipeline to detect chemosensory genes was implemented in Perl. Scripts are available on github (https://github.com/pbrec/CSGanalysis).

Only those ORFs that were reconstructed by both assemblers for a given species were included in preliminary candidate ORF sets for each gene family. Orthologs present in both species transcriptomes were identified using a reciprocal BLAST approach (see above). In order to prevent annotations of non gene family members and assembly artifacts, we mapped all sequence reads back to all loci in the preliminary candidate gene sets using Bowtie2 v2.0.4 [102] in the default global alignment mode. In this way all detected putative members of the chemosensory gene family sets were curated manually in Geneious v6.0.5 [103] taking sequence coverage and mapping accuracy into account.

In rare cases, high similarity between sequences detected in the *Euglossa* transcriptomes and the reference sequences allowed for subsequent manual identifications of homologs of the other species only assembled by one assembler. The resulting chemosensory gene family sets constituted the basis of all subsequent analyses.

### Phylogenetic analyses

For each chemosensory gene family we calculated a maximum likelihood (ML) phylogenetic gene-tree using RaxML v7.2.7 [104] to infer the potential genealogical histories of the candidate euglossine gene family members. On that account, gene family specific alignments of the protein sequences of all candidate euglossine and other known hymenopteran proteins of the same gene family (see Additional file 1: Table S3 for details) were produced using MAFFT v7.031b [105, 106] applying the L-INS-I algorithm with the --maxiterate option set to 1000 [107].

In order to find the model for amino acid sequence evolution that fits the data best, Prottest v3.2 [108] was applied on each alignment testing for all 120 models available. The proposed model (JTT + G for ORs; CpREV + I + G for GRs; LG + I + G for IRs, OBPs and CSPs) was used to infer an unrooted ML tree in RaxML. Ten independent ML searches on ten randomized parsimony trees were conducted to find the tree with the highest likelihood. Of these, the tree with the highest likelihood was chosen and bootstrap analyses with 1000 replicates were conducted. Then, the tree was rooted by a known outgroup or, for gene families lacking a known outgroup, by mid-point rooting (See Fig. 1 and Additional file 2: Figures S1-S4).

### Signatures of selection

We used the pooled read sets representing 40 males per species to infer sites fixed for differences between all detected orthologous NC and chemosensory genes with ≥10-fold mean per-base coverage in *Euglossa dilemma* and *E. viridissima*. Polymorphic sites were discarded since the nature of non-barcoded pooled RNA-Seq data prevents the reconstruction of individual haplotypes. Pre-processed reads were mapped onto the nucleotide sequences of the detected genes and the complete ORF together with 100 bp upstream and downstream the ORF was used as reference to allow inclusion of reads spanning the ORF and adjacent untranslated regions. For the mapping step global alignments with Bowtie2 were performed in the highly sensitive mode with -L set to 21 to adjust for the minimum read length.

A site was considered fixed if a minimum of 95 % of all reads spanning the site exhibited an identical nucleotide character at the specific site in the sequence of one species absent at the homologous site in the homologous sequence of the other species. Nucleotide characters were deemed present at a minimum of five reads, or five percent of all reads when higher than five, showing the respective nucleotide character at the site of interest. These restrictions were applied to counteract bias through probable sequencing errors. All pairs of orthologs showing fixed differences were saved as pairwise nucleotide sequence alignments and subsequently used for the maximum-likelihood estimation of pairwise *d*_*N*_*/d*_*S*_ ratios [109] using estimated transition to transversion ratios (model M1) in codeml of the PAML package v4.6 [110]. The *d*_*N*_*/d*_*S*_ ratios for each pairwise comparison were tested for significant deviations from a neutral model (*d*_*N*_*/d*_*S*_ = 1). Therefore, we conducted likelihood-ratio tests of the likelihood estimates for the M1 model and the likelihood estimated with *d*_*N*_*/d*_*S*_ fixed to one (model M0; ∆ = 2(ln(M1)-ln(M0)) with ∆ approximating a chi-square distribution with one degree of freedom).

### Prediction of ligand binding domains

Since non-synonymous mutations in the TM regions of ORs and in the S1 and S2 ligand binding domains of the IR related iGluRs can lead to differences in ligand binding affinities [12, 63–65, 67, 81, 82] we predicted TM topology and S1 and S2 domains for ORs and IRs, respectively, revealing patterns of positive selection.

For TM prediction we used online versions of TMpred (http://www.ch.embnet.org/software/TMPRED_form.html; [111]), TMHMM (http://www.cbs.dtu.dk/services/TMHMM/; [112]) and Topcons (http://topcons.net; [113]). The respective highest-ranking prediction of topologies was selected for each candidate OR and prediction tool. Non-synonymous substitutions were mapped onto the predicted receptor topologies and those mapping within TM regions supported by at least two of the three prediction tools were called TM substitutions. This step was necessary, since bioinformatic tools for TM prediction can deviate in their outputs for the same genes (*e.g.* [114]).

We aligned candidate *Euglossa* IRs and the conserved *Drosophila melanogaster* IR8a (DmelIR8a) receptor using MAFFT as described earlier to predict the S1 and S2 ligand binding domains. On that account, the known S1 and S2 ligand binding sites of DmelIR8a [42] were used to transfer annotations to the *Euglossa* IRs.

### Availability of supporting data

The data sets supporting the results of this article are included within the article and its additional files. Raw sequence reads are available at the NCBI Sequence Read Archive [SRA: SRX765918, SRA: SRX765888].

## Additional files

Additional file 1: Supplementary Tables S1-S6. (XLSX 3214 kb) Additional file 2: Supplementary Figures S1-S5. (PDF 3452 kb)

## Abbreviations

CSP: Chemosensory protein
Gb: Gigabases
GR: Gustatory receptor
HNDB: 2-hydroxy-6-nona-1,3-dienyl-benzaldehyde
iGluR: Ionotropic glutamate receptor
IR: Ionotropic receptor
ML: Maximum likelihood
NC: Non-chemosensory
OBP: Odorant-binding protein
OR: Odorant receptor
ORF: Open reading frame
TM: Transmembrane
