# Supplementary material for: Rapid evolution of chemosensory receptor genes in a pair of sibling species of orchid bees (Apidae: Euglossini)
Source: BMC Evol Biol. 2015 Aug 28;15:176. doi: 10.1186/s12862-015-0451-9 (PMC4552289; doi:10.1186/s12862-015-0451-9)
Supplement: Additional file 2: Supplementary Figures S1-S5. — (PDF 3452 kb) [file 12862_2015_451_MOESM2_ESM.pdf]

Supplementary Figures

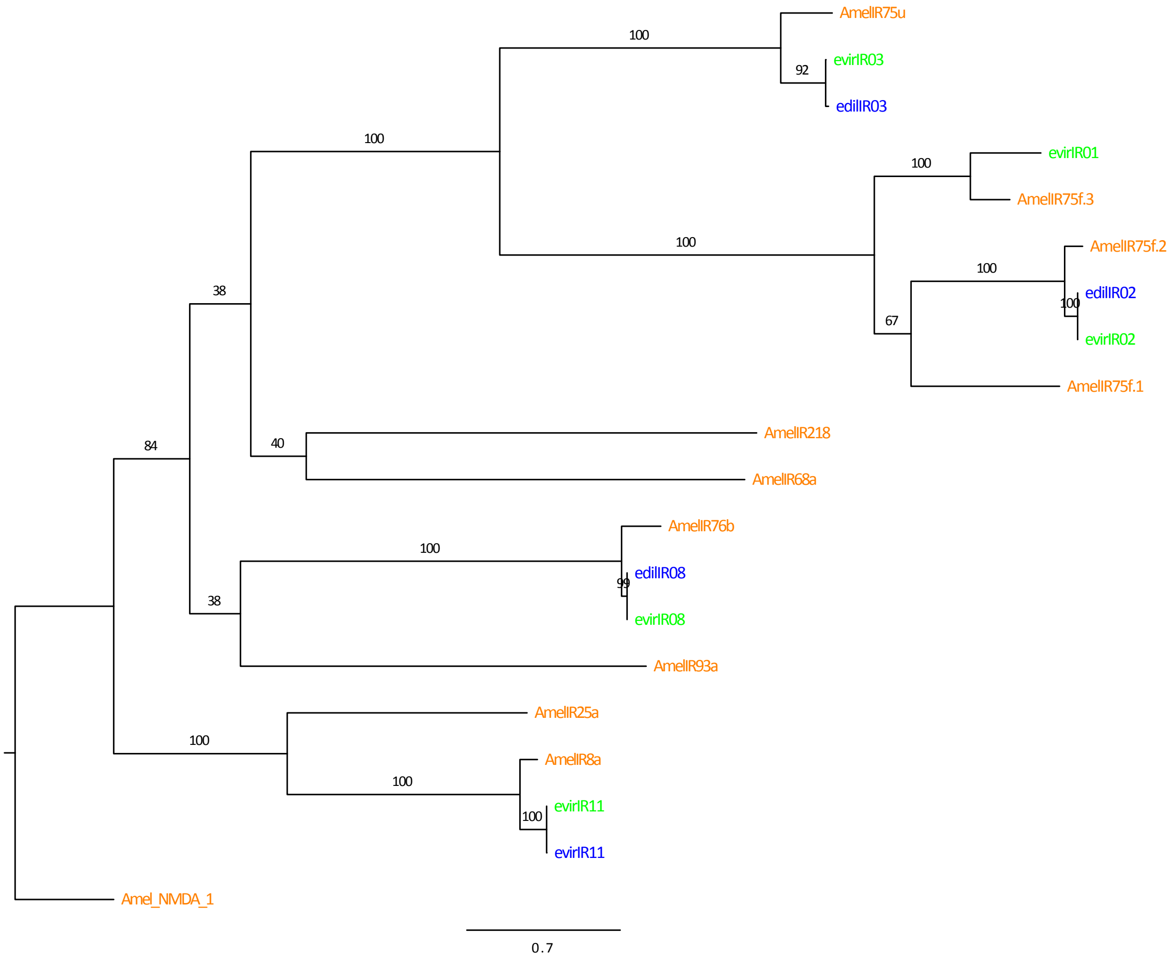

Figure S1. Phylogenetic relationships of the 4 candidate *Euglossa dilemma* (blue) and 5 *Euglossa viridissima* (green) Ionotropic Receptors to *Apis mellifera* (orange). This maximum likelihood tree was rooted by *A. mellifera* NMDA1. Numbers above branches show bootstrap-supports based on 1000 replicates.

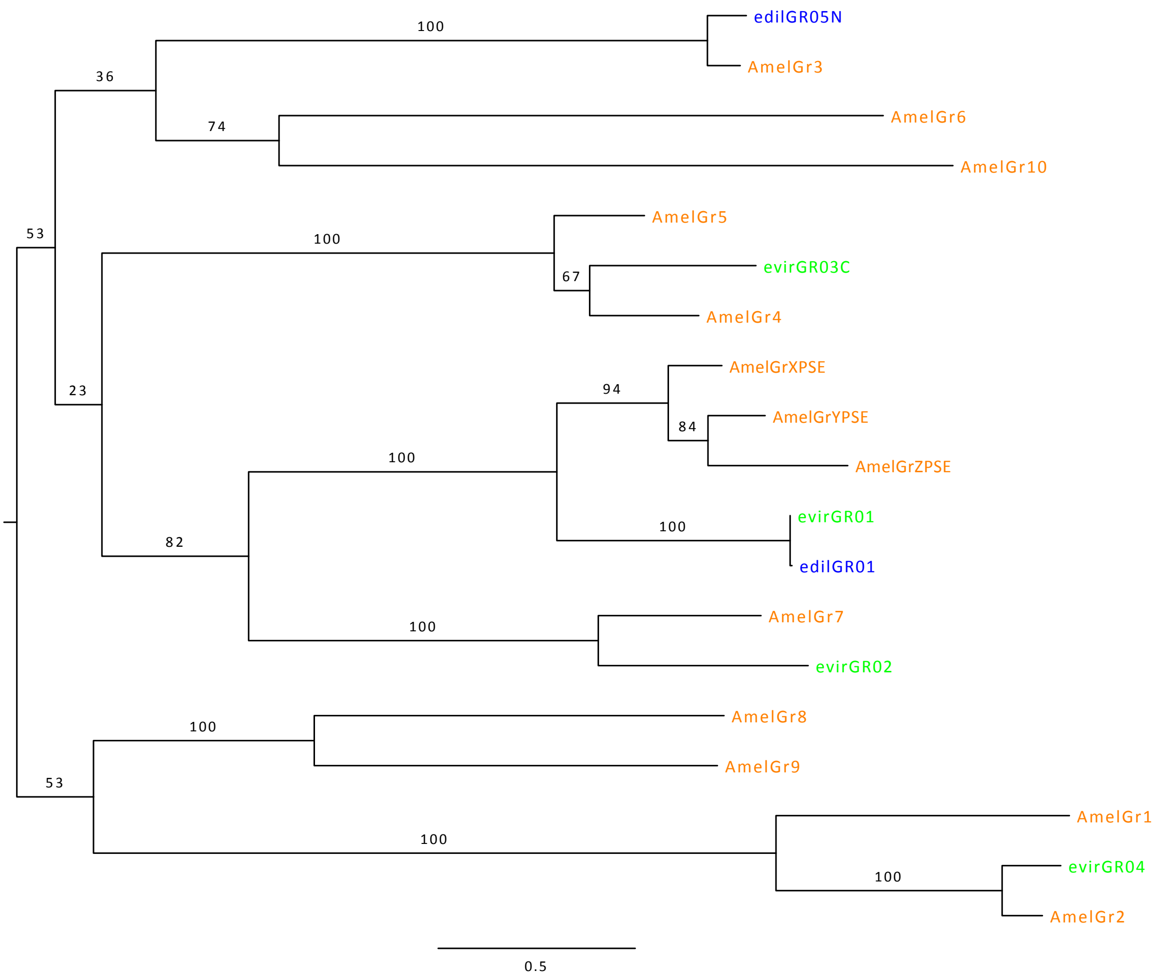

Figure S2. Phylogenetic relationships of the 2 candidate *E. dilemma* (blue) and 4 *E. viridissima* (green) Gustatory Receptors to *A. mellifera* (orange). This maximum likelihood tree was rooted at the midpoint in absence of a suitable outgroup. Numbers above branches show bootstrap-supports based on 1000 replicates. Symbols after the GR descriptors: C: C-terminus is missing, N: N-terminus is missing, PSE: pseudogene (after Robertson and Wanner 2006).

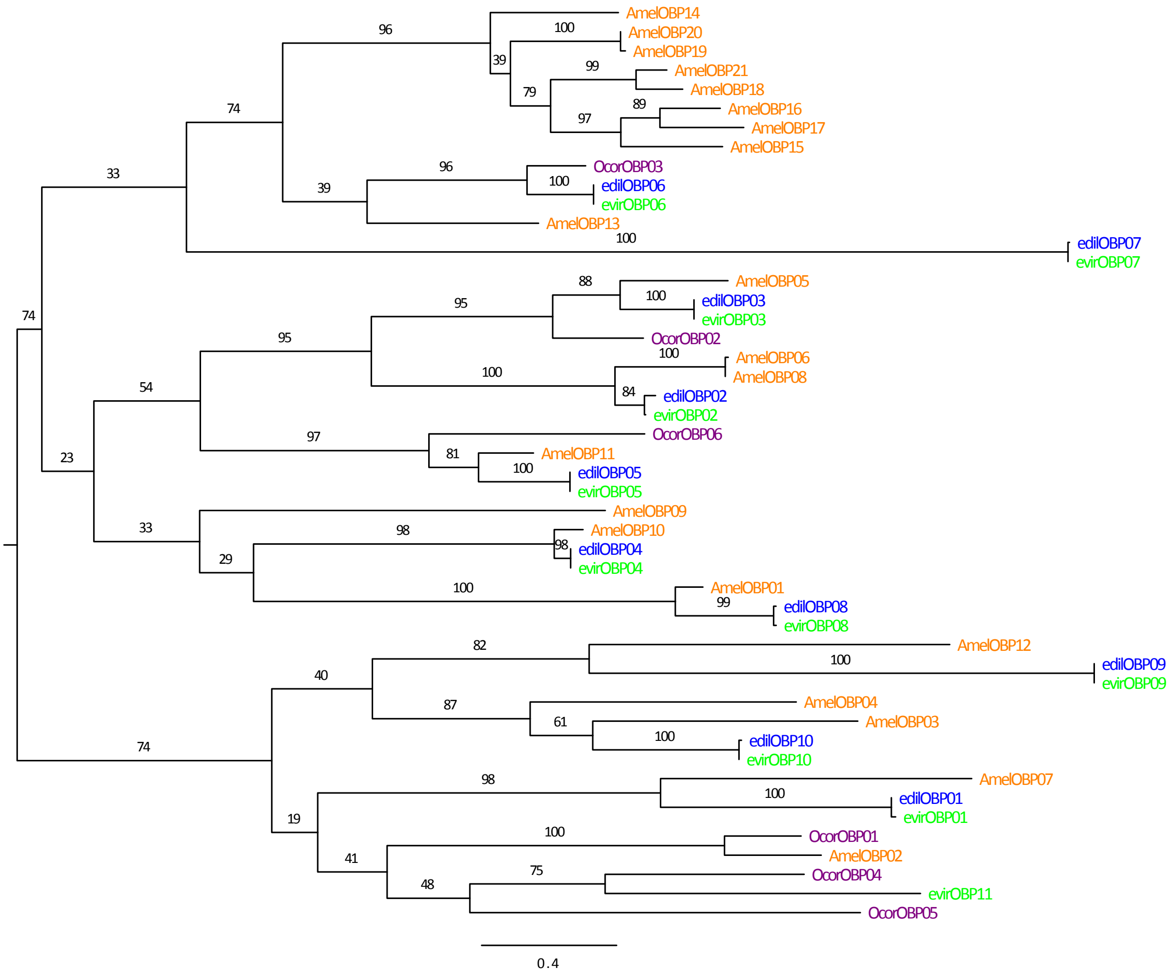

Figure S3. Phylogenetic relationships of the 10 candidate *E. dilemma* (blue) and 11 *E. viridissima* (green) Odorant Binding Proteins to *A. mellifera* (orange) and *Osmia cornuta* (purple). This maximum likelihood tree was rooted at midpoint in absence of a suitable outgroup. Numbers above branches show bootstrap-supports based on 1000 replicates.

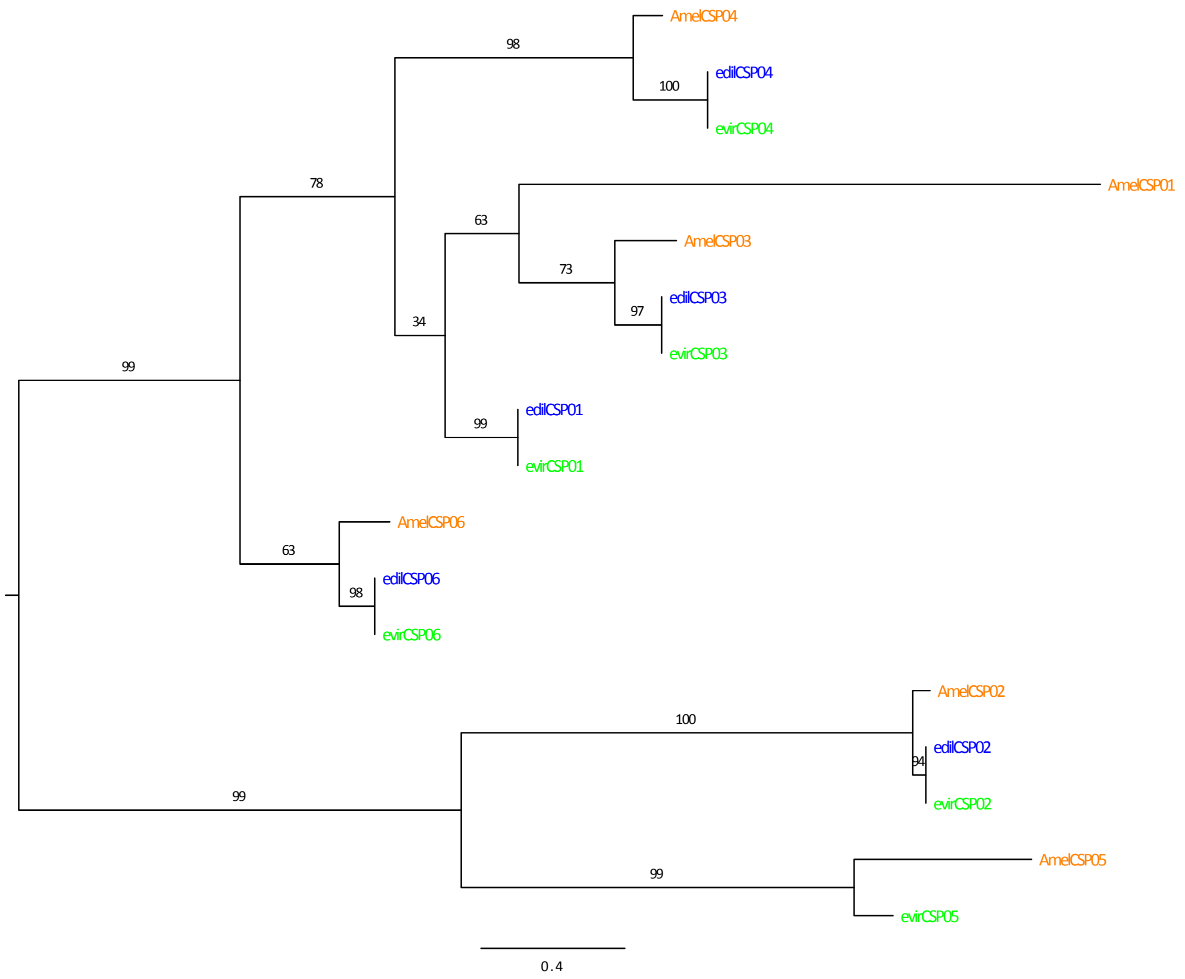

Figure S4 Phylogenetic relationships of the 5 candidate *E. dilemma* (blue) and 6 *E. viridissima* (green) Chemosensory Proteins to *A. mellifera* (orange) The maximum likelihood tree was rooted at midpoint in absence of a suitable outgroup. Numbers above branches show bootstrap-supports based on 1000 replicates.

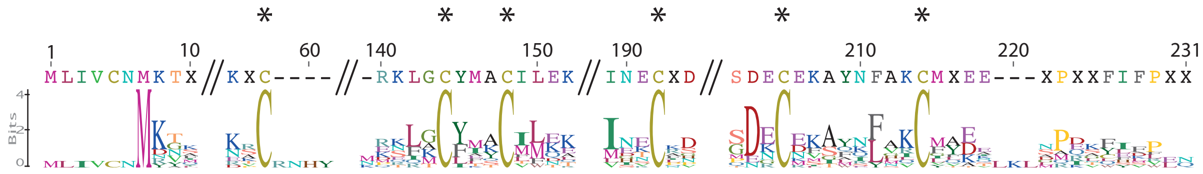

Figure S5 Sequence logo of the protein sequence alignment of all candidate *Euglossa* OBPs Relative sizes of letters indicate the frequency of an amino acid at the given site among the entire alignment. * indicates conserved cysteines present in all OBP sequences. Letters above the sequence logo represent the consensus sequence of the alignment at indicated positions.

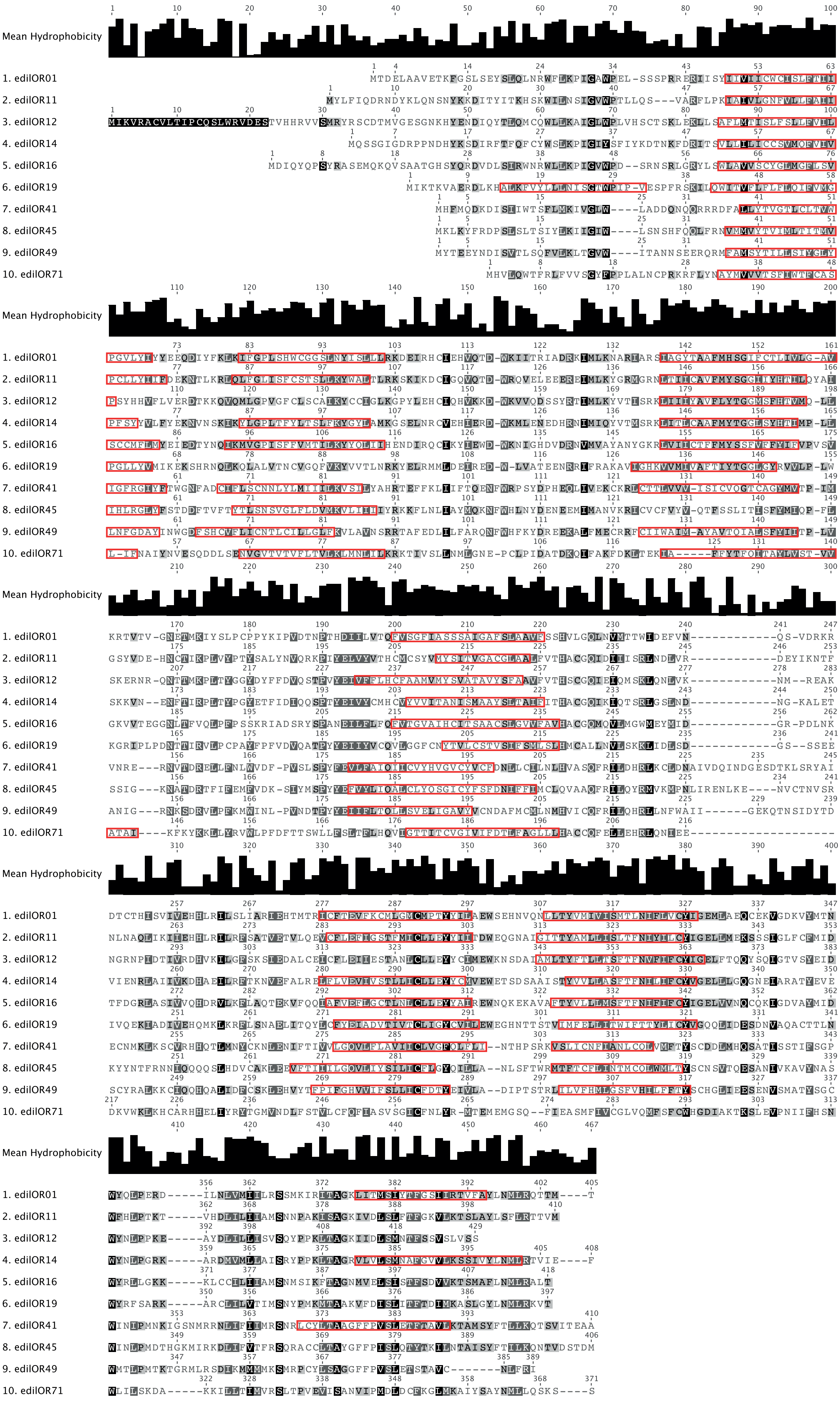
Mean Hydrophobicity
1. edilOR01
2. edilOR11
3. edilOR12
4. edilOR14
5. edilOR16
6. edilOR19
7. edilOR41
8. edilOR45
9. edilOR49
10. edilOR71

Figure S6 Distribution of transmembrane (TM) domains across Odorant Receptor (OR) sequences. All ORs showing $d_N/d_S>1$ represented by the *Euglossa dilemma* homologs (indicated by edilORXX) were aligned using MAFFT (see Materials and Methods) and consensus TM predictions were mapped onto the respective OR sequences (Red boxes; Supplementary Table S5). Amino acids are colored by the degree of conservation relative to amino acids at homologous sites in the alignment using a color gradient from white for unconserved to black for highly conserved residues. Mean hydrophobicity is indicated for each site in a barplot above the alignment. The Plot was generated in Geneious v5.4.6.
